# Combinatorial Natural Killer Cell–based Immunotherapy Approaches Selectively Target Chordoma Cancer Stem Cells

**DOI:** 10.1158/2767-9764.CRC-21-0020

**Published:** 2021-12-02

**Authors:** Austin T.K. Hoke, Michelle R. Padget, Kellsye P. Fabian, Anjali Nandal, Gary L. Gallia, Marijo Bilusic, Patrick Soon-Shiong, James W. Hodge, Nyall R. London

**Affiliations:** 1 Sinonasal and Skull Base Tumor Program, National Institutes on Deafness and Other Communication Disorders, NIH, Bethesda, Maryland.; 2 University of North Carolina at Chapel Hill, School of Medicine, Chapel Hill, North Carolina.; 3 Laboratory of Tumor Immunology and Biology, Center for Cancer Research, NCI, NIH, Bethesda, Maryland.; 4 Department of Neurosurgery, Johns Hopkins University School of Medicine, Baltimore, Maryland.; 5 Department of Otolaryngology-Head and Neck Surgery, Johns Hopkins University School of Medicine, Baltimore, Maryland.; 6 Genitourinary Malignancies Branch, Center for Cancer Research, NCI, NIH, Bethesda, Maryland.; 7 ImmunityBio, Culver City, California.

## Abstract

Chordoma is a rare tumor derived from notochord remnants that has a propensity to recur and metastasize despite conventional multimodal treatment. Cancer stem cells (CSC) are implicated in chordoma's resistant and recurrent behavior; thus, strategies that target CSCs are of particular interest. Using *in vitro* cytotoxicity models, we demonstrated that anti-programmed death ligand 1 (anti–PD-L1; N-601) and anti-EGFR (cetuximab) antibodies enhanced lysis of chordoma cells by healthy donor and chordoma patient NK cells through antibody-dependent cellular cytotoxicity (ADCC). Treatment of NK cells with an IL15 superagonist complex (N-803) increased their cytotoxicity against chordoma cells, which was further enhanced by treatment with N-601 and/or cetuximab. PD-L1–targeted chimeric antigen receptor NK cells (PD-L1 t-haNKs) were also effective against chordoma cells. CSCs were preferentially vulnerable to NK-cell killing in the presence of N-601 and N-803. Flow cytometric analysis of a chordoma CSC population showed that CSCs expressed significantly more NK-activating ligand B7-H6 and PD-L1 than non-CSCs, thus explaining a potential mechanism of selective targeting. These data suggest that chordoma may be effectively targeted by combinatorial NK cell–mediated immunotherapeutic approaches and that the efficacy of these approaches in chordoma and other CSC-driven tumor types should be investigated further in clinical studies.

**Significance:**

Combinatory immunotherapy using NK-mediated approaches demonstrates robust antitumor activity in preclinical models of chordoma and selectively targets chordoma CSCs.

## Introduction

Chordoma is a rare, slow growing, locally invasive primary bone tumor thought to develop from embryonic notochord remnants, resulting in its predilection for the axial skeleton. The annual incidence of chordoma in the United States is roughly 300 cases per year (1). In adults, approximately half of these tumors arise in the sacrum, with the remainder occurring in the spheno-occipital region of the skull base (35%) and mobile spine (15%; ref. 2). These anatomic regions are often in close proximity to critical neurovascular structures, thus posing technical challenges and morbidity to the current treatment mainstays of surgical resection and radiotherapy. Furthermore, chordomas are resistant to cytotoxic chemotherapy (3). Challenges to conventional therapeutic approaches are evidenced by rates of local recurrence as high as 68% and metastases in up to 40% of cases (2, 4). Median overall survival after diagnosis is approximately 5–7 years and decreases in patients with recurrent or metastatic disease (2, 5). Efforts to explore the application of immunotherapy in chordoma have led to clinical trials investigating targeted agents such as therapeutic vaccines (6, 7) and existing immunotherapeutic mainstays such as immune checkpoint blockade (NCT03173950, NCT02936102). However, these trials target chordoma patients with advanced disease and have yet to deliver durable responses. In the setting of recurrent and metastatic chordoma, survival rates are further compromised, and patients suffer increased morbidity from multiple rounds of surgical resection and radiation. Novel immunotherapy approaches hold promise to fill this unmet clinical need in chordoma.

Natural killer (NK) cells are a key focus of modern anti-cancer immunotherapy research. They uniquely kill multiple adjacent cells, do not depend on antigen presentation or specificity for their activity, and coordinate immune responses with antibodies and T cells.

Prior work has demonstrated the efficacy of avelumab (anti-programmed death ligand 1; PD-L1), the only immune checkpoint inhibitor that also promotes antibody-dependent cellular cytotoxicity (ADCC) with NK cells, in preclinical models of chordoma (8). N-601 is an anti–PD-L1 mAb and structural analog of avelumab that has not been previously investigated. N-803 (formerly ALT-803) is a clinical grade IL15 superagonist complex known to induce proliferation and activation of NK- and T-cell immune compartments (9, 10). Notable antitumor effects and augmentation of NK cell–mediated cytotoxicity (11–14) by N-803 in preclinical cancer models has supported several clinical trials focused on N-803 as monotherapy (ref. 10; NCT03054909, NCT02099539, NCT01946789) and in combination with other immunotherapeutic agents (refs. 15, 16; NCT03853317, NCT03022825, NCT03127098). We hypothesized that combinatory immunotherapy approaches with N-601 and N-803 can stimulate antitumor NK-cell responses in preclinical models of chordoma to a degree unmatched by prior efforts.

Cancer stem cells (CSC), or tumor-initiating cells (TIC), are a subpopulation of malignant cells that can drive tumorigenesis and disease relapse (17–20). This cell type is implicated in tumor heterogeneity and resistance to chemotherapy and radiation. CSCs have been identified in myeloid malignancies, glioblastoma, and cancers of the breast, colon, pancreas, and skin (21). The presence of a CSC population may be in part responsible for chordoma's recurrent nature and resistance to standard therapeutic approaches (8, 22, 23). Novel therapeutic options should be evaluated in their activity against chordoma CSCs, but the efficacy of immunotherapy against chordoma CSCs remains largely unstudied. Prior work has demonstrated that residential chordoma CSCs can be identified by coexpression of surface markers CD15, CD24, and CD133 (8, 22). Herein, we aim to further characterize this cellular subpopulation and investigate the potential of combinatorial NK cell–mediated approaches to selectively target chordoma CSCs. This study also intends to provide preclinical evidence to support future clinical investigation of combinatory NK cell–based immunotherapy approaches in chordoma that implement ADCC-mediating antibodies, NK-cell augmentation through cytokine agonism, and targeted adoptive NK-cell therapies.

## Materials and Methods

### Cell Lines and Culture

UM-Chor1 (clival) and JHC7 (sacral) were kindly provided by the Chordoma Foundation on approximately April 2, 2015 with short tandem repeat (STR) validation. U-CH1 (sacral), U-CH2 (sacral), UM-Chor5 (clival), and MUG-CC1 (clival) were obtained from ATCC on approximately July 20, 2020 with STR validation. All cell lines were passaged twice per week, used at a low (<30) passage number, and serially verified to be free of *Mycoplasma* infection using a two-step luminescence assay (Lonza Bioscience).

### Study Approval

Healthy donor peripheral blood mononuclear cells (PBMC) were obtained from the NIH Clinical Center Blood Bank (NCT00001846). Whole blood from a treatment-naïve chordoma patient was collected through a tissue procurement protocol (NCT03429036) between the NIH and Johns Hopkins University Hospital (Baltimore, MD). Whole blood from patients with chordoma receiving immuno-oncology therapy (NCT02383498, NCT03173950, NCT03349983, NCT01519817, NCT01772004, NCT02179515, NCT02536469) was obtained from collaborators at the NIH (Bethesda, MD). Each study was approved by the Institutional Review Board and informed written consent was obtained for enrollment on the respective institutional review board protocol. These studies were conducted in accordance with U.S. Common Rule.

### Immune Reagents and NK Cells

An anti–PD-L1 antibody (N-601), an IL15/IL15r superagonist (N-803), and the PD-L1–specific chimeric antigen receptor–engineered high-affinity NK cells (PD-L1 t-haNKs) were provided by ImmunityBio under a Cooperative Research and Development Agreement (CRADA) with the NCI of the NIH (Bethesda, MD). Cetuximab (Erbitux) was obtained from Lilly. Human IgG1 isotype control antibody and human anti-CD16 antibody were obtained from BioLegend.

PBMCs were isolated from chordoma patient whole blood using BD Bioscience Vacutainer CPT tubes according to the manufacturer's protocol. NK cells were isolated from PBMCs using the Miltenyi Biotec Human NK Cell Isolation (negative selection) Kit according to the manufacturer's protocol with >90% purity. NK cells were rested overnight in RPMI1640 media before being used as effector cells in assays.

For experiments using chordoma patient NK cells as effector cells, treatments undergone by patients prior to providing blood for this study are as follows: One patient, CP1(n), had a newly diagnosed clival chordoma and provided blood on the day of primary surgical resection. This patient had not received any other therapies (i.e., radiation, immunotherapy) prior to providing blood. A second patient, CP2(io), had a recurrent sacral chordoma and was previously treated with surgical resection, radiation, yeast brachyury vaccine (NCT02383498), anti–PD-1 mAb (NCT03173950), and modified vaccinia Ankara – Fowlpox brachyury vaccine (NCT03349983). A third patient, CP3(io), had a recurrent thoracic spine chordoma and was previously treated with surgical resection, radiation, yeast brachyury vaccine (NCT01519817), anti–PD-L1 mAb (NCT01772004), modified vaccinia Ankara–based vaccine modified to express brachyury and T-cell costimulatory signals (NCT02179515), anti-IL8 mAb (NCT02536469), and modified vaccinia Ankara – Fowlpox brachyury vaccine (NCT03349983).

### Flow Cytometric Analysis and FACS

For general cell surface marker analysis, chordoma cells were labeled with LIVE/DEAD 488 Fixable Green Dead Cell Stain Kit (Life Technologies, #L34970) according to the manufacturer's protocol. Cells were then washed and resuspended in FACS buffer (PBS + 1% BSA). Human Fc block (BD #564219) was added to 1 × 10^6^ cells and incubated for 10 minutes on ice. Cells were stained with HLA-A, B, C-APC (BioLegend #311410), PD-L1-BV421 (BD #563738), or EGFR-PE (BD #555997) and fixed with Cytofix (BD #554655). Marker expression was quantified by percent positive cells and mean fluorescence intensity (MFI). Flow cytometry was performed on BD FACSCanto (BD Biosciences) and analyzed using FlowJo v10.7.1 (TreeStar).

The CSC population in the UM-Chor1 cell line was identified as CD24^+^CD15^+^CD133^+^ cells using the following antibodies from BD Biosciences: CD24-BV711 (ML5), CD15-PE (6D4), and CD133-APC (W6D3). To stain for surface markers, PD-L1-BV605 (MPC11, BioLegend), MICA/B-PECy7 (6D4, BioLegend), HLA-A, B, C-BV605 (W6/32, BioLegend) and B7-H6-AlexaFluor700 (875001, R&D Systems) antibodies and their appropriate isotype controls were used. Flow cytometry was performed on BD LSRFortessa (BD Biosciences) and analyzed using FlowJo V.10.7.1 (TreeStar).

### Indium-111 NK-Cell Killing Assay

NK cells (effector cells) were isolated from healthy donor (HD) PBMCs or chordoma patient PBMCs as previously described. NK cells isolated from PBMCs were cultured overnight in the NK-cell media described above with or without 50 ng/mL of N-803. All NK cells were washed in the above-described media after overnight incubation. Cryopreserved PD-L1 t-haNK cells were thawed on the day of the experiment and washed two times in the previously described NK-cell media before being adjusted to the desired concentration and plated.

Chordoma cells (target cells) were labeled with ^111^In (10 μL/100,000 cells). NK cells (healthy donor, chordoma patient, or PD-L1 t-haNKs) were added to wells at various effector-to-target (E:T) ratios, depending on the experiment. Specific E:T ratios are indicated in the figure legends. All E:T ratios are 20:1 unless indicated otherwise. After 20 hours, assay plates were centrifuged at 1,500 rpm and supernatants were quantified for the presence of ^111^In using a PerkinElmer WIZARD2 Automatic Gamma Counter. Spontaneous ^111^In release was determined by incubating target cells without effector cells, and complete lysis was determined by incubating target cells with 0.05% Triton X-100. Experimental lysis was standardized using the following equation: Percent lysis = [(experimental cpm − spontaneous cpm)/(complete cpm − spontaneous cpm)] × 100. Negative control values in each bar graph represent spontaneous lysis of target cells without effector cells. All experiments were carried out in technical triplicate, with each individual experiment being repeated at least three separate times unless otherwise specified.

### Flow-based NK-Cell Killing Assay

UM-Chor1 cells were labeled with CellTrace Violet Proliferation dye (Thermo Fisher Scientific) and cocultured for 4 hours with healthy donor-derived NK cells that had been treated with N-803 overnight. Afterwards, the cocultures were stained with Live/Dead Fixable Aqua stain (Thermo Fisher Scientific) and the CSC marker antibodies described above. Flow cytometry was performed on BD LSRFortessa (BD Biosciences) and analyzed using FlowJo V.10.7.1 (TreeStar). The CSC and non-CSC populations were downsampled using FlowJo such that each cellular subtype would have similar numbers of cells in the histogram.

### Statistical Analysis

Experiments were carried out in technical triplicate with each experiment being repeated at least three times unless otherwise specified due to limitations by the NK-cell donor. Comparisons in NK-cell killing models of two independent groups were performed using a Student *t* test with a two-tailed distribution at a confidence level of 95%. Comparison of more than two independent groups were performed using one-way ANOVA with Tukey multiple comparisons test. All error bars indicate SEM. Test results are reported as P values with a significance cutoff set at *P* < 0.05. All analyses were performed using GraphPad 9.0 software (GraphPad Software Inc.).

### Data Availability

The data generated in this study are available upon request from the corresponding author.

## Results

### Chordoma Cell Lines from Distinct Anatomic Locations are Susceptible to Lysis by Healthy Donor NK Cells

Preclinical and clinical evidence indicate that tumor immune microenvironments are shaped by tissue of origin and anatomic location, thus impacting disease progression and response to immunotherapies (24). Given the anatomically distinct locations along the axial skeleton where chordoma arises and previously reported differences in survival between cranial chordomas and other locations (25), we first sought to determine whether there is a difference in target surface marker expression between three chordoma cell lines derived from clival chordomas (UM-Chor1, MUG-CC1, UM-Chor5) and three lines from sacral chordomas (JHC7, U-CH1, U-CH2; refs. 26–29, 30). Chordoma tissue samples and cell lines have been shown to express PD-L1 (8, 31–33, 34) and EGFR (35–37), both of which are surface antigens with clinically available mAbs that mediate ADCC with NK cells (38, 39). All six chordoma cell lines examined expressed HLA-A, B, C/MHC-1, PD-L1, and EGFR as assessed by percent positive cells and MFI (Table 1). There were no statistically significant differences between clival and sacral chordoma cell lines in expression of HLA-A, B, C (% positivity *P =* 0.18, MFI *P =* 0.67), PD-L1 (% positivity *P =* 0.89, MFI *P =* 0.89), or EGFR (% positivity *P =* 0.55, MFI *P =* 0.77).

**TABLE 1:** Six chordoma cell lines; established from 3 from clival tumor patients (UM-Chor1, MUG-CC1, UM-Chor5), and 3 sacral tumor patients (JHC7, U-CH1, U-CH2) were analyzed by flow cytometry to quantify expression surface markers MHC-I/HLA-A,B,C, PD-L1, and EGFR. Cell surface expression of each marker is reported in % positive cells and MFI.

		HLA-A,B,C/MHC-I		PD-L1		EGFR	
Cell Line	Derivation	% positive	MFI	% positive	MFI	% positive	MFI
**UM-Chor1**	66-year-old male Primary clival chordoma	100	7444	7.2	552	86.9	879
**MUG-CC1**	72-year-old male Primary clival chordoma	90.8	1781	7.4	1723	24.5	2859
**UM-Chor5**	<20-year-old male Primary clival chordoma	97.6	4439	10.2	1167	46.0	1427
**JHC7**	61-year-old female Primary sacral chordoma	91.0	2042	10.9	558	52.1	1094
**U-CH1**	56-year-old male Recurrent sacral chordoma	92.6	4782	7.1	1600	26.4	2619
**U-CH2**	72-year-old female Recurrent sacral chordoma	90.9	4301	7.5	1487	40.7	2164

**FIGURE 1:**
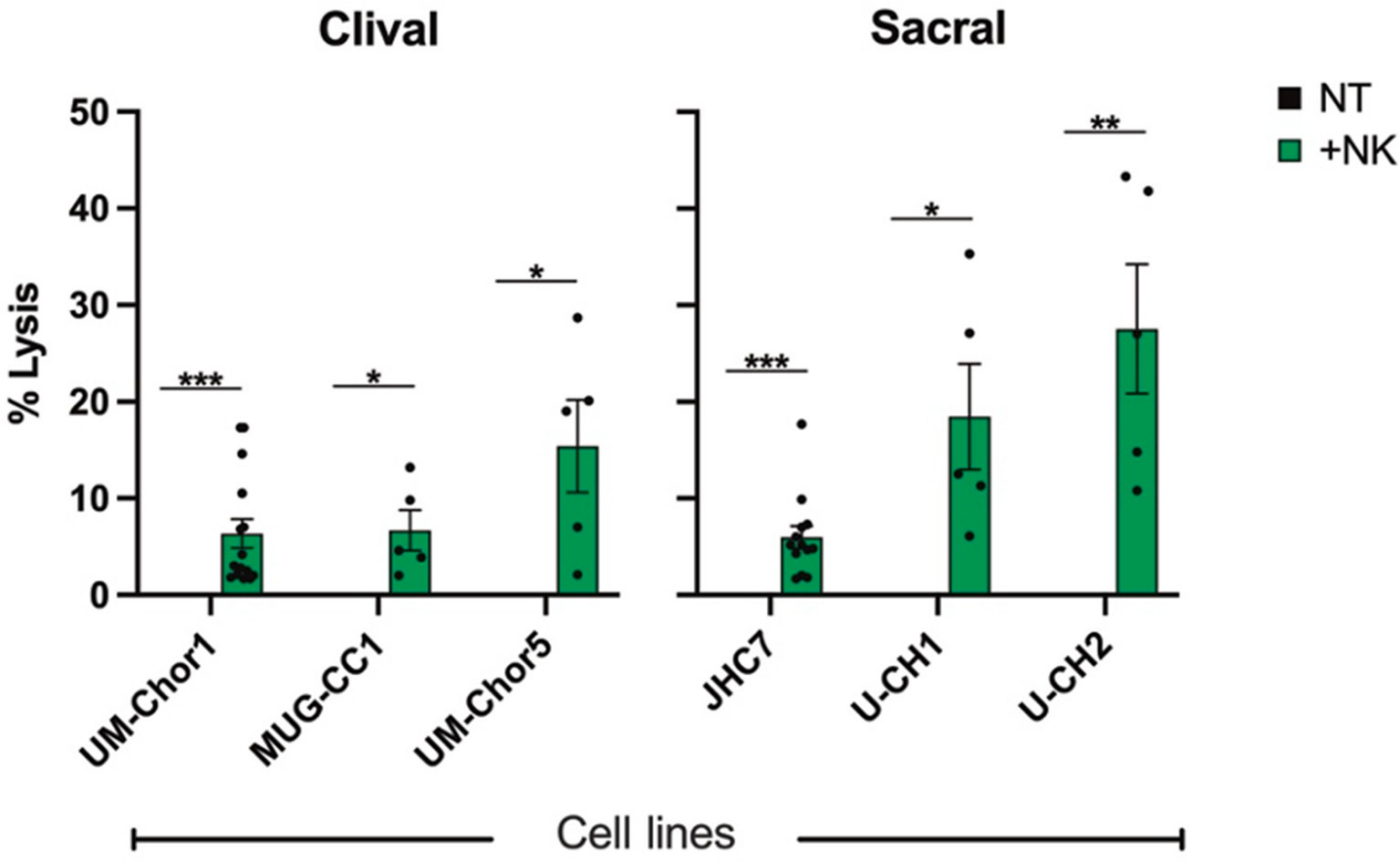
Anatomically distinct chordoma cell lines are susceptible to lysis by healthy donor natural killer (NK) cells. Chordoma cell killing by 25 healthy NK cell donors as determined by ^111^In-release killing assays. Each data point represents a distinct healthy NK cell donor. All E:T ratios are 20:1. Statistical analyses were done by Student's t-test. *P d 0.05, **P d 0.01, ***P d 0.001, ****P d 0.0001. Results shown are the means ± SEM of at least five independent experiments.

Next, we interrogated the susceptibility of chordoma cell lines to lysis by healthy donor (HD) NK cells and determined whether the site of anatomic derivation had any significant impact on this susceptibility. Consistent with prior studies using *in vitro* NK-cell killing assays (8, 39), the average NK cell–mediated lysis of chordoma cell lines was significantly higher than spontaneous lysis nontreated (NT) controls but remained low overall (average: 10.7%; SEM: 1.5%), highlighting the potential for augmentation in NK cell–mediated lysis of chordoma cells (Fig. 1). There was no significant difference between NK cell–mediated lysis of clival and sacral chordoma cell lines (*P =* 0.09), indicating that anatomic derivation of chordoma cell lines does not alter sensitivity to lysis by NK cells.

### Anti–PD-L1 Antibody (N-601) Enhances Killing of Chordoma Cells Through ADCC

**FIGURE 2:**
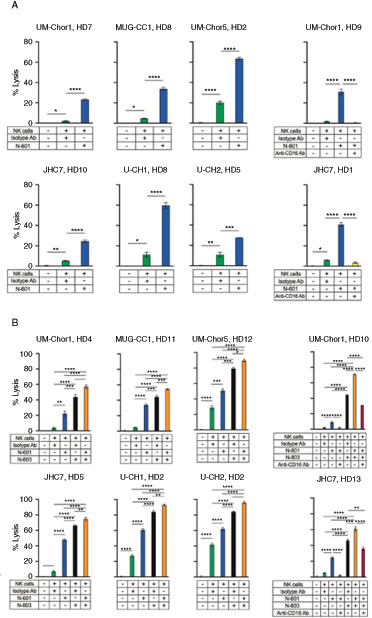
Chordoma cell susceptibility to lysis by healthy donor NK cells is enhanced by N-601 (anti-PD-L1), N-803 (IL15 superagonist), and combination treatment with N-601 and N-803. Six chordoma cell lines (UM-Chor1, MUG-CC1, UM-Chor5, JHC7, U-CH1, U-CH2) were used as targets for healthy donor NK cells in ^111^In-release killing assays. **A,** N-601-mediated ADCC assays were performed by co-incubating chordoma cells with N-601 or isotype control antibody. NK cells were treated with anti-CD-16 antibody where indicated. **B,** Combination treatment assays were performed by co-incubating chordoma cells with N-601 or isotype control antibody and treating NK cells with N-803 and/or anti-CD-16 antibody where indicated. All E:T ratios are 20:1. Statistical analyses were done by one-way ANOVA with Tukey's multiple comparisons test. *P d 0.05, **P d 0.01, ***P d 0.001, ****P d 0.0001. Results shown are the means ± SEM of technical triplicate measurements and are representative of three independent experiments.

Our group has demonstrated that a clinically relevant anti–PD-L1 antibody, avelumab, enhances NK cell–mediated lysis of chordoma cells via ADCC (8). We used a recently developed anti–PD-L1 antibody, N-601, to perform similar *in vitro* ADCC killing assays with healthy donor NK cells as effector cells and chordoma cell lines as target cells. N-601 significantly increased NK cell lysis of 6 of 6 chordoma cell lines compared with isotype control antibody (Fig. 2A). The average increase in NK cell–mediated tumor cell lysis by N-601 in >20 independent experiments was 6.2-fold (*P* d 0.0001) compared with isotype control antibody.

Given that N-601 is a structural homolog of avelumab and maintains the human Fc element, we hypothesized ADCC to be the primary mechanism through which N-601 enhanced NK cell–mediated killing of chordoma cells. Blocking CD16 on NK cells with a neutralizing antibody before coculture with chordoma cells entirely abrogated the NK-cell killing of tumor cells (*P* d 0.0001; Fig. 2A and B), indicating that N-601 enhanced NK-cell lysis of chordoma cells through an ADCC-dependent mechanism.

### IL15/IL15r Superagonist (N-803) Enhances NK-Cell Killing of Chordoma Cells, and Combinatorial Treatment with Anti–PD-L1 Antibody (N-601) and N-803 Further Enhances Cytotoxicity

N-803 is a humanized IL15 superagonist that stimulates and expands NK cells and T cells (9, 10). While this immunostimulatory complex has demonstrated antitumor efficacy as a monotherapy and in combination against multiple cancer types in clinical studies (10, 15) NCT03054909, NCT02099539, NCT01946789, NCT03853317, NCT03022825, NCT03127098), it has yet to be explored in chordoma. NK cells treated with N-803 were significantly more cytolytic against chordoma cell lines compared with untreated NK cells (Fig. 2B). The average increase in tumor cell lysis by N-803 treatment of NK cells in >20 independent experiments was 13.6-fold (*P* d 0.0001) compared with untreated controls.

We hypothesized that combination treatment of chordoma cells with N-601 and NK cells with N-803 would further enhance tumor cell lysis by NK cells compared with monotherapy treatments. This combinatorial approach, which has not yet been evaluated in the literature, resulted in additional cytotoxic effect on chordoma cells when compared with untreated controls (Fig. 2B). The average increase in tumor cell lysis by combinatory treatment with N-601 and N-803 in >10 independent experiments was 15.5-fold (*P* d 0.0001) compared with untreated controls. Combination treatment with N-601 and N-803 significantly enhanced NK-cell killing of 6 of 6 chordoma cell lines compared with monotherapy with N-601 (*P* d 0.0001) or N-803 (*P* d 0.05; Fig. 2B).

To evaluate the contribution of ADCC to NK cell–mediated killing of chordoma cells when N-601 and N-803 are combined, NK cells were treated with a CD16 neutralizing antibody before coculture with chordoma cells. This resulted in attenuated killing when compared with both combination treatment (*P* d 0.0001) and N-803 alone (UM-Chor1 *P* d 0.0001; JHC7 *P =* 0.0056; Fig. 2B). This result suggests that in the presence of N-601, ADCC still plays a critical role in the lysis of chordoma cells by N-803–activated NK cells.

### Anti-EGFR Antibody (Cetuximab) Enhances Killing of Chordoma Cells Through ADCC and Combinatorial Treatment with Cetuximab and IL15/IL15r Superagonist (N-803) Further Enhances Cytotoxicity

**FIGURE 3:**
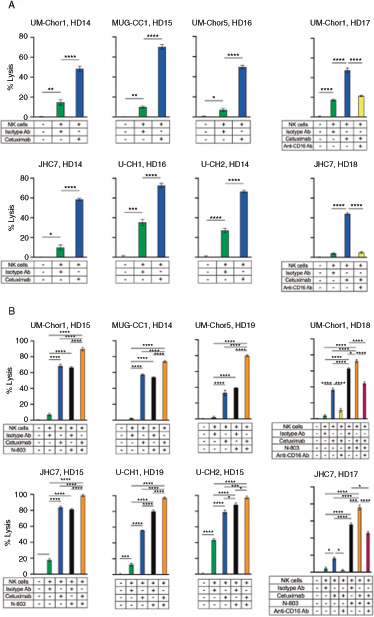
Chordoma cell susceptibility to killing by healthy donor NK cells is enhanced by cetuximab (anti-EGFR) and combination treatment with N-803 (IL15 superagonist) and cetuximab. Six chordoma cell lines (UM-Chor1, MUG-CC1, UM-Chor5, JHC7, U-CH1, U-CH2) were used as targets for healthy donor NK cells in ^111^In-release killing assays. **A,** Cetuximab-mediated ADCC assays were performed by co-incubating chordoma cells with N-601 or isotype control antibody. NK cells were treated with anti-CD-16 antibody where indicated. **B,** Combination treatment assays were performed by co-incubating chordoma cells with cetuximab or isotype control antibody and treating NK cells with N-803 and/or anti-CD-16 antibody where indicated. All E:T ratios are 20:1. Statistical analyses were done by one-way ANOVA with Tukey's multiple comparisons test. *P d 0.05, **P d 0.01, ***P d 0.001, ****P d 0.0001. Results shown are the means ± SEM of technical triplicate measurements and are representative of three independent experiments.

Previous work by our group has also demonstrated that an anti-EGFR mAb, cetuximab, mediates NK-cell lysis of chordoma cells via ADCC (39). These findings were confirmed and extended here with additional clival and sacral chordoma cell lines, as we showed that cetuximab increased NK-cell lysis of 6 chordoma cell lines compared with isotype control antibody (Fig. 3A). The average increase in tumor cell lysis by cetuximab in >20 independent experiments was 9.3-fold (*P* d 0.0001) compared with isotype control antibody. Furthermore, neutralization of CD16 on NK cells with a blocking antibody resulted in abrogation of tumor cell lysis (*P* d 0.0001; Fig. 3A and B) which is consistent with previous findings that cetuximab enhances NK-cell lysis of chordoma cells via ADCC (39).

Given that N-803 enhanced ADCC-mediated lysis of chordoma cells in the presence of N-601, we hypothesized that combinatory treatment with cetuximab and N-803 would similarly result in greater tumor cell lysis than monotherapy approaches. This therapeutic combination, not yet studied in chordoma, resulted in additional cytotoxic effect against chordoma lines when compared with untreated controls as follows (Fig. 3B). The average increase in tumor cell lysis by combinatory treatment with cetuximab and N-803 in >10 independent experiments was 24.2-fold (*P* d 0.0001) compared with untreated controls. Combination treatment with cetuximab and N-803 significantly enhanced NK-cell killing of 6 of 6 chordoma cell lines compared with cetuximab monotherapy (*P* d 0.001) and N-803 monotherapy (*P* d 0.05).

CD16 neutralization on NK cells attenuated killing when compared with combination treatment (*P* d 0.0001) and monotherapy with N-803 (UM-Chor1 *P* d 0.0001; JHC7 *P =* 0.01; Fig. 3B), indicating that cetuximab-mediated ADCC remains essential for increased tumor cell lysis when cetuximab and N-803 are combined.

### Doublet Treatment of Chordoma Cells with Anti–PD-L1 (N-601) and Anti-EGFR (Cetuximab) Antibodies Further Enhances Sensitivity to Lysis by IL15 Superagonist (N-803)-activated NK Cells

**FIGURE 4:**
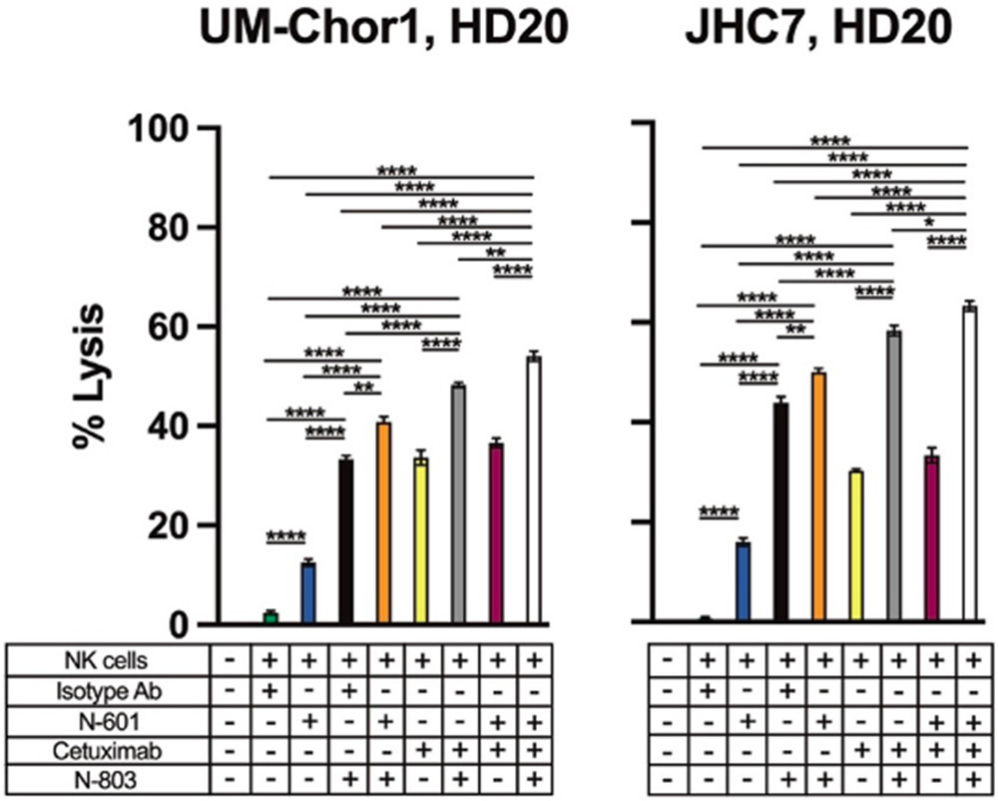
Doublet treatment of chordoma cells with N-601 (anti-PD-L1) and cetuximab (anti-EGFR) further enhances sensitivity to lysis by N-803 (IL15 superagonist)-activated NK cells. Two chordoma cell lines (UM-Chor1, JHC7) were used as targets for healthy donor NK cells in ^111^In-release killing assays. Chordoma cells were co-incubated with isotype control antibody, N-601, and/or cetuximab and NK cells were treated with N-803 where indicated. All E:T ratios are 10:1 to prevent excess tumor cell lysis and allow for the assessment of incremental enhancement by triple combination therapy. Statistical analyses were done by one-way ANOVA with Tukey's multiple comparisons test. *P d 0.05, **P d 0.01, ***P d 0.001, ****P d 0.0001. Results shown are the means ± SEM of technical triplicate measurements and are representative of three independent experiments.

Flow cytometric analysis of 6 chordoma cell lines revealed higher EGFR expression than PD-L1 expression (Table 1; Supplementary Fig. S1A). Furthermore, PD-L1^+^ cells also tended to be EGFR^+^, as evidenced by the robust cytotoxicity of cetuximab-mediated ADCC (Fig. 3). However, we identified small subpopulations of cells that appeared to be PD-L1^+^/EGFR^−^ (Supplementary Fig. S1A), thus theoretically posing additional antitumor activity by combining therapeutic antibodies targeting both surface antigens. To model this doublet ADCC-promoting antibody treatment strategy with N-803–enhanced effector cells (Supplementary Fig. S1B), two chordoma cell lines were treated with N-601 and cetuximab before being targeted by N-803–activated NK cells. This combination approach proved to be marginally more effective than the next best therapeutic combination of cetuximab and N-803 (UM-Chor1 *P =* 0.003; JHC7 *P =* 0.03; Fig. 4).

### Chordoma Patients’ NK Cells Mediate Significant Lysis of Chordoma Cells and are Enhanced with Anti–PD-L1 Antibody (N-601), Anti-EGFR Antibody (Cetuximab), and IL15/IL15r Superagonist (N-803)

**FIGURE 5:**
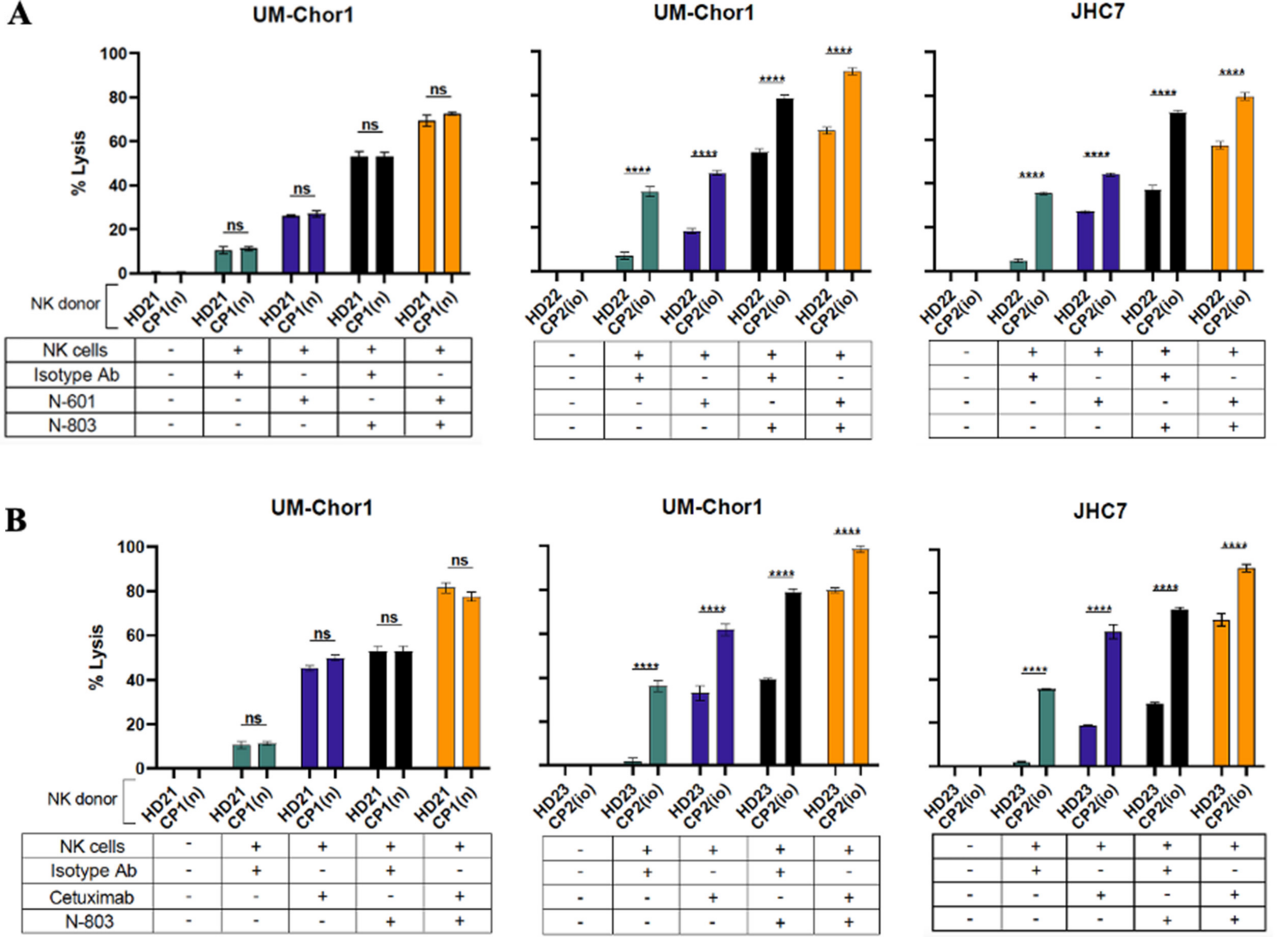
Chordoma patients’ NK cells mediate significant lysis of chordoma cells and are enhanced with N-601 (anti-PD-L1), cetuximab (anti-EGFR), and N-803 (IL15 superagonist). Two chordoma cell lines (UM-Chor1, JHC7) were used as targets for healthy donor NK cells or chordoma patient NK cells in ^111^In-release killing assays. Combination treatment assays were performed by co-incubating chordoma cells with N-601 (**A**), cetuximab (**B**), or isotype control antibody **(A and B**) where indicated. Healthy donor NK cells (**A and B**), treatment-naïve chordoma patient (CP1(n)) NK cells (**A and B**), or previously-treated chordoma patient (CP2(io)) NK cells (**A and B**) were treated with N-803 where indicated and used as effectors. All E:T ratios are 20:1. Statistical analyses were done by one-way ANOVA with Tukey's multiple comparisons test. *P d 0.05, **P d 0.01, ***P d 0.001, ****P d 0.0001. Results shown are the means ± SEM of technical triplicate measurements and are representative of two independent experiments with recurrent chordoma patients and one experiment with a treatment-naïve chordoma patient.

Patients with cancer have alterations in immune cell function (40), thus we next sought to determine whether NK cells from patients with chordoma are responsive to ADCC-mediating antibodies and N-803 exposure. We isolated NK cells from three patients with chordoma. Similar to NK cells from healthy donors, NK cells from 3 of 3 patients with chordoma demonstrated enhanced cytotoxicity after exposure to N-601 (Fig. 5A), cetuximab (Fig. 5B), N-803 (Fig. 5A and B), and combination therapy (*P* d 0.05; Fig. 5A and B). This finding indicates that NK cells in patients with chordoma are not inherently impaired in their ability to lyse chordoma cells and be enhanced by ADCC and IL15 superagonism.

Interestingly, NK cells from 2 of 3 patients with chordoma exhibited significantly increased cytotoxic activity at baseline compared with healthy donor controls, and this persisted after treatment with N-601, cetuximab, N-803, and combination therapy (*P* d 0.0001). These patients, labeled CP(io), had recurrent chordoma and had undergone various treatment modalities (refer to Materials and Methods) prior to providing blood for this study (Fig. 5A and B; Supplementary Fig. S2). The patient with NK-cell activity similar to that of healthy donors, labeled CP1(n), had a primary chordoma and had not been treated prior to providing blood for the study (Fig. 5A and B).

### PD-L1 t-haNK Cells Induce Lysis of Chordoma Cells

**FIGURE 6:**
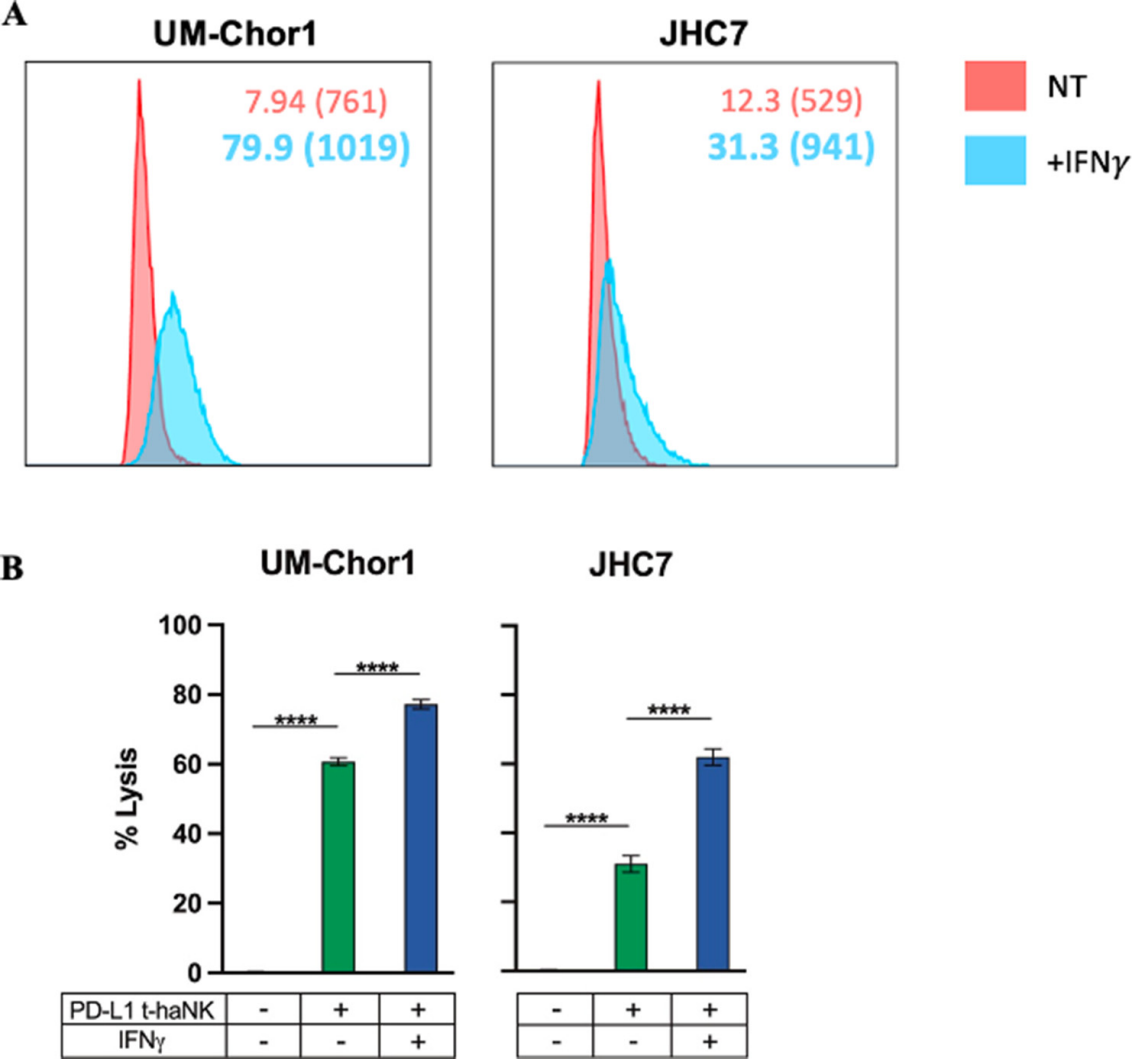
PD-L1 t-haNK cells induce lysis of chordoma cell lines through a PD-L1-mediated mechanism. Two chordoma cell lines (UM-Chor1, JHC7) were used as targets for PD-L1 t-haNK cells in ^111^In-release killing assays. **A,** Chordoma cells were analyzed by flow cytometry for PD-L1 expression after 24-hour co-incubation with IFNγ on the same day as killing assays. Cell surface expression of PD-L1 quantified by flow cytometry is reported in % positive cells and MFI. Bold values denote an increase of > 30% relative to untreated control cells. **B,** Chordoma cells were co-incubated with IFNγ or left untreated as controls for 24 hours before being used as targets for PD-L1 t-haNK cells. All E:T ratios are 20:1. Statistical analyses were done by one-way ANOVA with Tukey's multiple comparisons test. *P d 0.05, **P d 0.01, ***P d 0.001, ****P d 0.0001. Results shown are the means ± SEM of technical triplicate measurements and are representative of three independent experiments.

As a potential alternative clinical option for PD-L1–positive tumors, we interrogated the sensitivity of chordoma cell lines to lysis by human NK cells that have been genetically engineered to express the high-affinity CD16 receptor, endoplasmic reticulum–retained IL2, and a CAR-specific against PD-L1 (41, 42). The *in vitro* antitumor efficacy of these PD-L1–targeted high-affinity natural killer (PD-L1 t-haNK) cells against human cancer cell lines is dependent on PD-L1 expression levels on tumor cells (20). Here, we hypothesized that treatment of chordoma cell lines with IFNγ would upregulate PD-L1 expression, thus increasing their sensitivity to lysis by PD-L1 t-haNK cells. Our study showed that IFNγ increased PD-L1 expression (both % positive cells and MFI) on 2 of 2 chordoma cell lines (Fig. 6A) and in turn, enhanced the susceptibility of these cell lines to lysis by PD-L1 t-haNK cells (*P* d 0.0001; Fig. 6B).

Despite containing the high-affinity CD16 receptor, it has been shown that tumor cell lysis by PD-L1 t-haNK cells is not enhanced by therapeutic antibodies that induce ADCC, such as avelumab or cetuximab (20). Previous interrogation of this finding revealed that PD-L1 t-haNK killing mediated through the CAR occurs more readily than killing through ADCC (20). Similarly, treatment of PD-L1 t-haNK cells with N-803 does not enhance their cytotoxic function, as this engineered NK-cell line does not express the IL15 receptor (20). Here, we confirm that PD-L1 t-haNK lysis of chordoma cell lines is not enhanced by cotreatment with N-601, cetuximab, or N-803 (Supplementary Fig. S3).

### Anti–PD-L1 Antibody (N-601)–mediated ADCC and IL15/IL15r Superagonist (N-803) Activation of NK Cells Enhance Lysis of Chordoma CSCs

**FIGURE 7:**
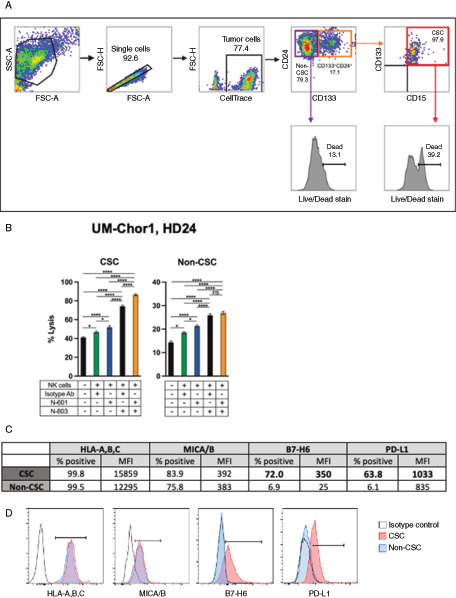
Targeting of chordoma CSCs through N-601 (anti-PD-L1)-mediated ADCC by N-803 (IL15 superagonist)-activated NK cells. CellTrace Violet-stained UM-Chor1 cells were used as targets for healthy donor NK cells in a flow cytometry-based killing assay. **A,** CSC and non-CSC subpopulations were identified using the gating strategy described. **B,** UM-Chor1 cells were co-incubated with N-601 or isotype control antibody and NK cells were treated with N-803 where indicated. CSC and non-CSC cell death through N-601-mediated ADCC by untreated or N-803-treated NK cells were evaluated via flow cytometry using a live/dead fixable cell stain exclusion method. (**C and D**) Flow cytometry was used to quantify expression of HLA-A,B,C, MICA/B, B7-H6, and PD-L1 expression on CSC and non-CSC UM-Chor1 cells. Cell surface expression of each marker is reported in % positive cells and MFI. Bold values denote an increase of > 20% when comparing CSC vs. non-CSC. All E:T ratios are 5:1. Statistical analyses were done by one-way ANOVA with Tukey's multiple comparisons test. *P d 0.05, **P d 0.01, ***P d 0.001, ****P d 0.0001. Results shown are the means ± SEM of technical quadruplicate measurements and are representative of two independent experiments.

CSCs are a subpopulation of cells within certain tumor types that play a significant role in tumorigenesis, metastasis, and resistance to therapy (18, 19, 43) and should therefore be a primary target of anticancer therapy. We have previously reported that exposure of chordoma cells to IFNγ increases PD-L1 expression on the cell surface, augmenting anti–PD-L1 antibody-mediated ADCC (8). Here, we investigated the susceptibility of chordoma CSCs by N-803–activated NK cells and anti–PD-L1–driven ADCC. Flow cytometry was utilized to distinguish the CSCs from the non-CSCs and to determine the degree of cell death in the two compartments using a viability exclusion dye. As defined in previous studies (8, 22), we designated the UM-Chor1 CSCs as cells that coexpress the surface markers CD15, CD24, and CD133 (Fig. 7A). Similar to our data in Figs. 2A, 4, 5A, and 5C, UM-Chor1 cells were susceptible to N-601–mediated ADCC as evidenced by increased cell death in both CSC and non-CSC populations compared with isotype control (*P* d 0.05; Fig. 7B). Likewise, activating NK cells with N-803 improved their cytotoxicity against both CSCs and non-CSCs when compared with untreated controls (*P* d 0.0001). A combinatory approach with N-601 and N-803 further enhanced NK-cell lysis of CSC cells (*P* d 0.0001), while cell death of non-CSCs by N-803 pretreated NK cells remained the same with or without N-601 (*P =* 0.46; Fig. 7B).

To elucidate the preferential NK-cell killing of UM-Chor1 CSCs over non-CSCs in the presence of N-601 and N-803, we used flow cytometry to examine the expression of NK ligands and PD-L1 on the two cellular subpopulations (Fig. 7C and D). CSCs and non-CSCs showed comparable expression of MICA/B, a ligand for the activating receptor NKG2D, and HLA-A, B, C/MHC class I, which binds NK-inhibitory receptors. However, CSCs expressed B7-H6, a ligand for the NK-activating receptor NKp30, at higher levels than the non-CSCs (>10-fold increase in % positive cells and MFI). Furthermore, compared with non-CSCs, the CSCs demonstrated elevated expression of PD-L1, both as percent positive cells and MFI (>10-fold increase in % positive cells, >20% increase in MFI; Fig. 7C and D). This upregulation of an NK-activating ligand and PD-L1 in the UM-Chor1 CSC population may sensitize CSCs to lysis by NK cells in a combinatory treatment paradigm with N-601 and N-803.

## Discussion

Chordoma is a devastating malignancy with resistance to traditional anticancer treatment modalities such as radiation and chemotherapy, thus necessitating the interrogation of novel treatment approaches. Prior foundational work has characterized the expression of PD-L1 (31–33, 34) and EGFR (35, 36, 37) in chordoma tumor specimens, thus setting the stage for novel targeted immunotherapeutic approaches to be interrogated. Numerous mAbs targeting PD-L1 and EGFR have been generated and approved to treat cancer. However, only one such antibody for each target molecule (PD-L1, avelumab; EGFR, cetuximab) retains the native human Fc region, enabling it to inhibit the desired signaling pathway and promote ADCC with NK cells. N-601 is a novel anti–PD-L1 structural analog of avelumab that also maintains the native human Fc region for ADCC coordination. Here, we capitalize on this unique and dynamic antibody feature and demonstrate that both N-601 and cetuximab enhance chordoma cell sensitivity to lysis by NK cells through an ADCC-dependent mechanism in 6 of 6 chordoma cell lines. (Figs. 2A and 3A).

N-803 (formerly ALT-803) is a clinical-grade IL15 superagonist complex known to induce proliferation and activation of NK and T-cell immune compartments (9, 10, 44). No preclinical or clinical studies have investigated the activity of N-803 in chordoma. Here for the first time, we show that N-803 significantly enhances NK-cell cytotoxic effect on 6 of 6 chordoma cell lines (Figs. 2B and 3B). Furthermore, combinatory treatment with an ADCC-mediating antibody (N-601, cetuximab) and N-803 enabled superior tumor cell lysis compared with monotherapy with any single agent (Figs. 2B and 3B). Flow cytometric analysis of chordoma cell lines (Supplementary Fig. S2A) supported a potential benefit in doublet antibody treatment with both N-601 and cetuximab in combination with N-803 activation of NK cells (Fig. 4). This is the first chordoma study demonstrating the efficacy of combinatorial immunotherapy with ADCC-mediating antibodies and an IL15 superagonist complex in an NK cell–based treatment paradigm. These data provide the preclinical rationale for the clinical investigation of immunotherapeutic combinations with NK-cell effectors in the treatment of chordoma, and also support future studies exploring the translation of such combinations to mediate T-cell activation against chordoma.

The failure of conventional cancer therapies, such as chemotherapy and radiation, in the chordoma setting may be partially due to CSCs (45, 46). While non-CSCs are susceptible to anticancer therapeutics, treatment-resistant CSCs can self-renew and maintain tumor growth. Harnessing NK-cell activity is a promising anti-CSC approach because NK cells are not affected by the downmodulation of antigen processing and presentation machinery molecules observed in CSCs (47). In addition, studies have shown that CSCs from various cancer types are susceptible to IL15-activated NK cells and this targeting was associated with increased expression of NK ligands on the surface of CSCs (48, 49). Here, we show that lysis of UM-Chor1 CSCs and non-CSCs by NK cells is improved in the presence of N-601 (Fig. 7C). When NK cells were activated by N-803, lysis of both tumor cell populations was further enhanced. This finding may be partially attributed to the MICA and MICB (a ligand for NK-activating receptor NKG2D) expression in both CSCs and non-CSCs, and to the expression of B7-H6 (ligand for the NK-activating receptor NKp30) in the CSC subpopulation (Fig. 7B). Combinatory treatment with N-601 and N-803 was effective at further enhancing lysis of the CSC population, which may be attributed to upregulation of PD-L1 in the CSCs compared with non-CSCs (Fig. 7B). Although CSCs make up only a minority subset within chordoma cell lines (8, 22), our study illustrates a preferential killing of CSCs compared with non-CSC in the described combinatory treatment strategy. A more comprehensive interrogation of chordoma CSC and non-CSC NK ligand expression is needed to better understand the driving mechanisms behind the observed differential target lysis. Moreover, differential NK-cell killing of the two cellular subpopulations may be further elucidated by sorting for CSCs and non-CSCs and coincubating equal numbers of cells with NK cells at increasing E:T ratios (20). This immunotherapy approach may be applicable to other tumor types with identifiable CSC populations and warrants further investigation.

Cancer induces a systemic immunosuppressive environment through altering the function of cellular and noncellular immunity (40). To support the translation of this proposed immunotherapy framework into the clinical setting, we included NK cells from three patients with chordoma and compared their cytotoxic function to healthy donor NK cells both at baseline and in the presence of ADCC-mediating antibodies and N-803. Chordoma patients’ NK cells were reflective of healthy donor NK cells throughout this study in their positive response to treatment with N-601, cetuximab, and/or N-803 (Fig. 5A–C). This result, though limited in sample size, suggests that chordomas do not impair NK-cell cytotoxic function or response to therapeutic enhancement with ADCC-mediating antibodies or IL15 superagonism. Identification of two patients with chordoma with enhanced NK-cell cytotoxicity (Fig. 5B and C) prompted interrogation of these patients’ clinical histories. These two patients had undergone surgical resections, multiple rounds of radiation, and participated in various previous immunotherapy protocols (refer to Materials and Methods) prior to providing blood for this study, while the patient with nonenhanced NK-cell activity had not received any prior therapy before providing study blood. Interestingly, neither of the patients with enhanced NK-cell function had received any therapy in at least 3 months before providing blood for this study. These results may suggest that prior therapy in patients with chordoma, likely one of the immunomodulating agents listed previously, can impart a sustained impact on the cytotoxic activity of NK cells without compromising their ability to respond to treatment with ADCC-mediating antibodies and N-803. Such sustained NK-cell responses to anticancer therapeutics have yet to be defined in the medical literature and the mechanism remains unknown. Conclusions drawn from this interesting finding are limited by sample size and lack of patient NK cells before and after treatment with immunotherapy, but the replicated response in two separate patients with chordoma warrants further investigation in a controlled clinical trial setting.

While this study focuses on ADCC-mediating antibodies and NK-cell antitumor activity, the agents investigated here are uniquely dynamic due to their ability to also stimulate T-cell responses. First, T cells comprise a much larger percentage of the lymphocyte population than do NK cells. N-601 is an immune checkpoint inhibitor, which has the established primary function of galvanizing T-cell antitumor immunity. The effect of immune checkpoint blockade on T cells, and not the coordination of ADCC with NK cells, underlies the encouraging progress brought forth by various checkpoint inhibitors and is evidenced by the fact that avelumab is the only checkpoint inhibitor that induces ADCC. Moreover, IL15 superagonism with N-803 also bolsters T-cell proliferation and activity (9, 10, 44). The true impact of combinatory immunotherapy with N-601 and N-803 in chordoma will require preclinical studies with antigen-specific T-cell models, but may also be demonstrated by future clinical studies with appropriate T-cell immune correlate investigation.

Limitations to this study include the *in vitro* nature of our preclinical models, which is related to poor NK-cell engraftment in PBMC humanized triple knockout NSG mice as well as other barriers to experimentation with murine chordoma xenograft models. As previously discussed, mechanistic drivers of the enhanced cytotoxic activity of NK cells from patients with recurrent chordoma with prior treatments remains unknown and should be investigated further in a controlled clinical trial setting. Also previously mentioned, the dynamic ability of this combinatory model to stimulate T-cell responses through immune checkpoint blockade and IL15 superagonism are not explored here. Future preclinical and clinical studies using more comprehensive biologic models will be critical in evaluating the antitumor efficacy of the above proposed treatment framework.

## Supplementary Material

Supplementary Data Supplementary Data
