# Supplementary material for: Combinatorial Natural Killer Cell–based Immunotherapy Approaches Selectively Target Chordoma Cancer Stem Cells
Source: Cancer Res Commun. 2021 Dec 2;1(3):127–39. doi: 10.1158/2767-9764.CRC-21-0020 (PMC9236084; doi:10.1158/2767-9764.CRC-21-0020)
Supplement: Supplementary Data [file crc-21-0020-s01.docx]

Supplementary Material for

**Combinatorial natural killer cell-based immunotherapy approaches**

**selectively target chordoma cancer stem cells**

Austin T.K. Hoke et al.

**The PDF file includes:** Figs. S1 to S3

**Fig. S1A**. **Chordoma cell lines express PD-L1 and EGFR with small subpopulations of PD-L1+/EGFR-** **cells.** Six chordoma cell lines were analyzed by flow cytometry. Representative flow cytometric scatter plots of PD-L1 and EGFR co-expression are shown here.

*
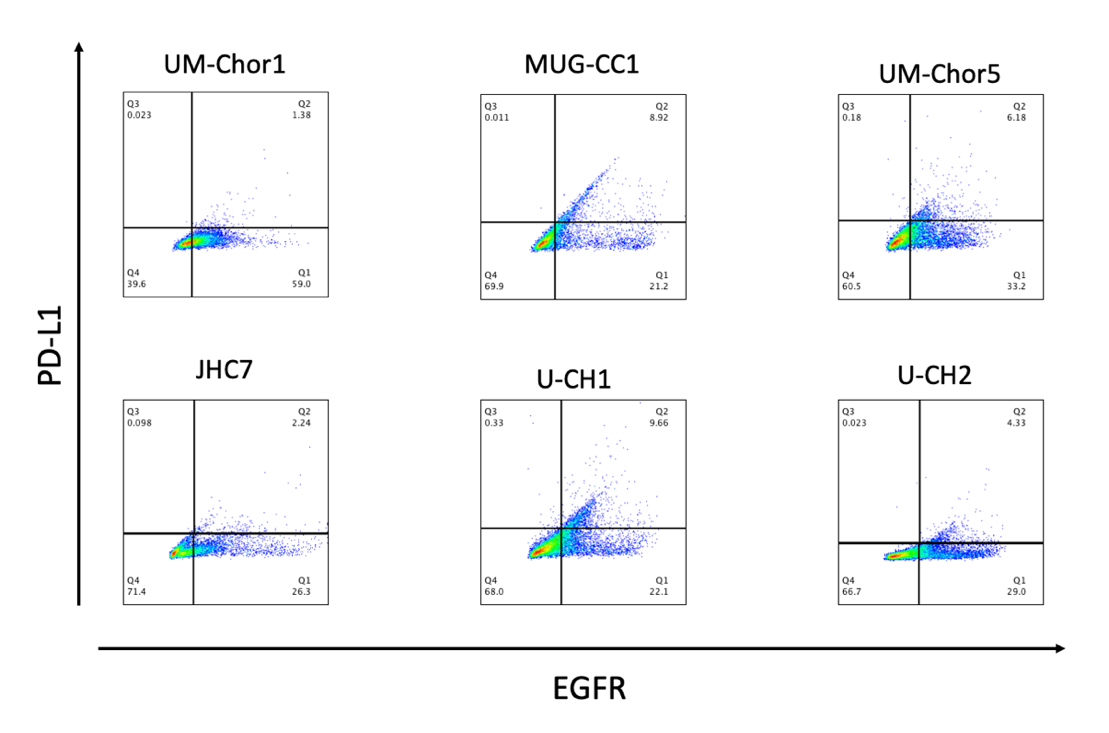
*

**
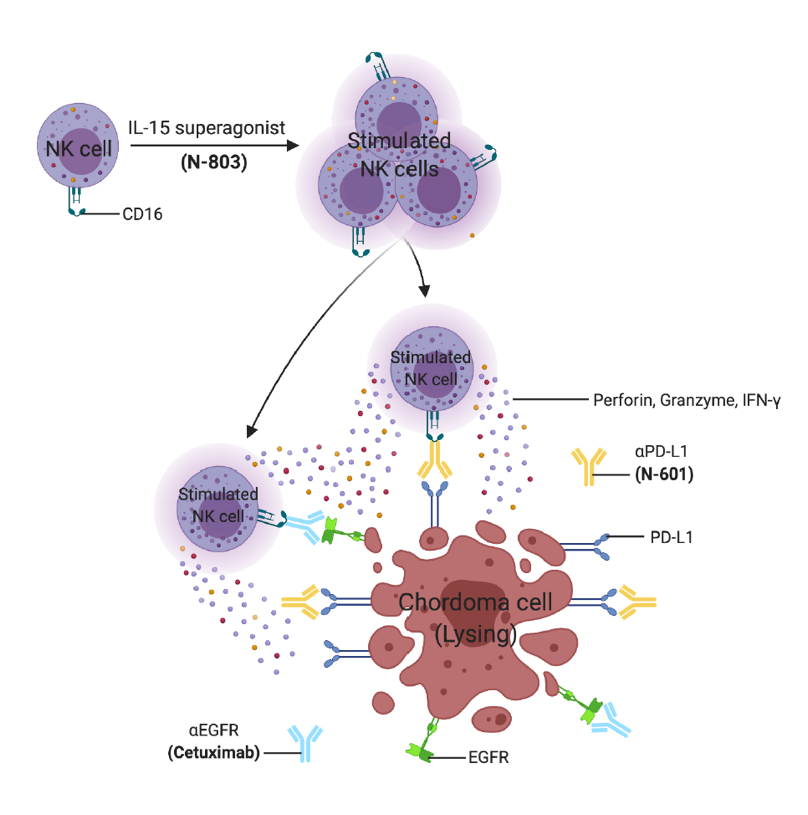
Fig. S1B. Schematic of N-803-enhanced NK cells binding to ADCC-mediating antibodies on a chordoma cell, inducing tumor cell lysis.**

**Fig. S2. Previously treated chordoma patients’ NK cells demonstrate enhanced cytotoxicity and are further enhanced with N-601 (anti-PD-L1), cetuximab (anti-EGFR), and N-803 (IL-15 superagonist).**

**
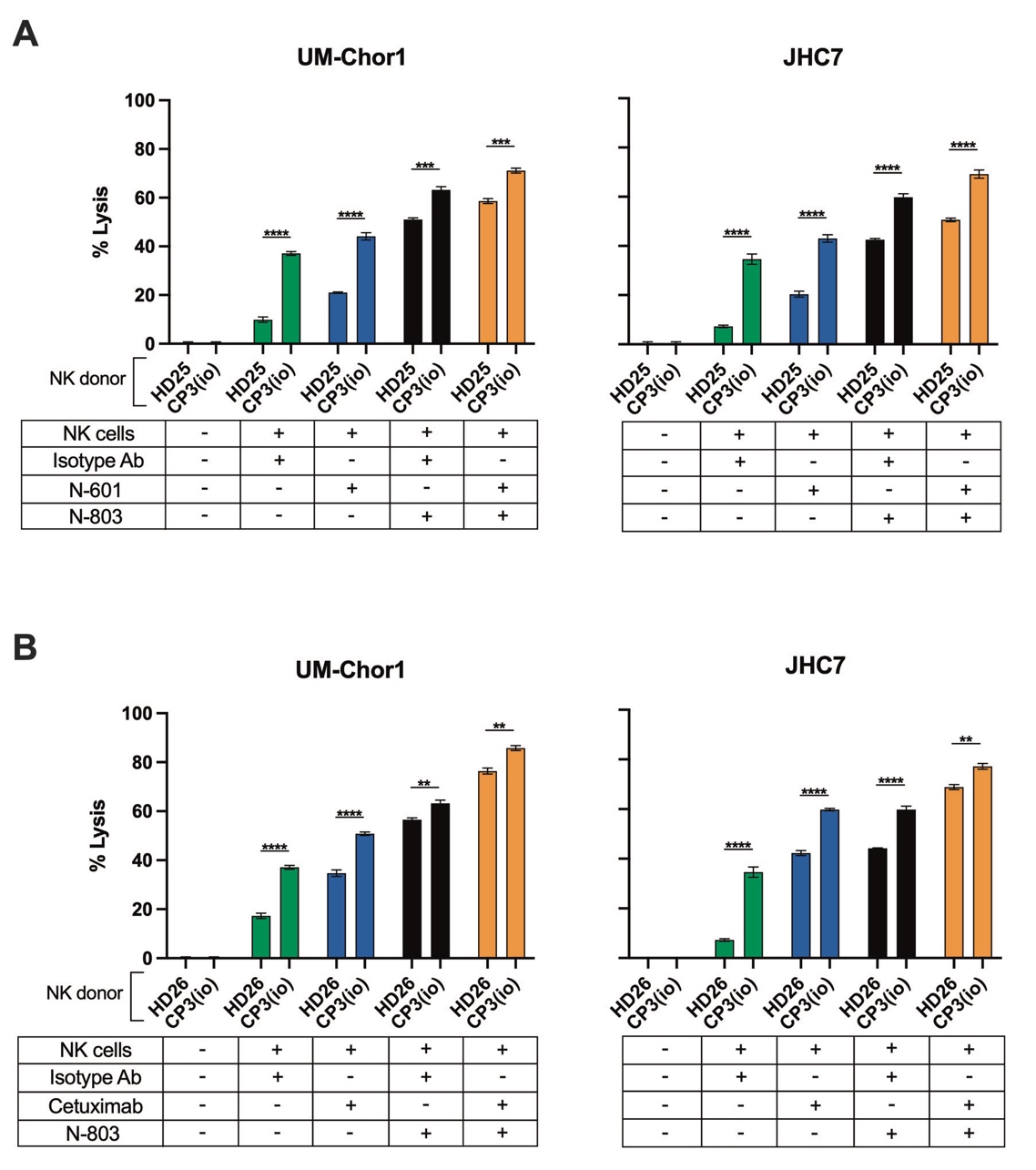
**

**Fig. S3. Lysis of chordoma cells by PD-L1 t-haNK cells is not enhanced by ADCC-mediating antibodies (N-601, cetuximab) or N-803.** UM-Chor1 cells were used as target cells for PD-L1 t-haNK cells in ^111^In-release killing assays. Target cells were co-incubated with N-601 and/or cetuximab where indicated. PD-L1 t-haNK effector cells were treated with N-803 where indicated.

**
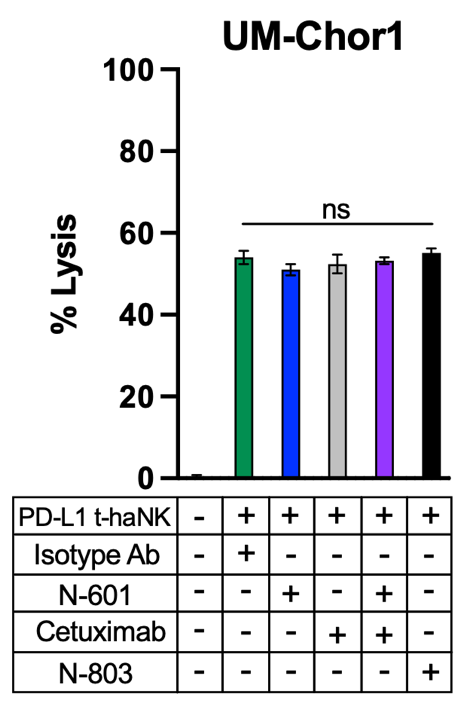
**
